# Relationship between gene expression patterns from nasopharyngeal swabs and serum biomarkers in patients hospitalized with COVID-19, following treatment with the neutralizing monoclonal antibody bamlanivimab

**DOI:** 10.1186/s12967-022-03345-3

**Published:** 2022-03-18

**Authors:** Jonathan T. Sims, Josh Poorbaugh, Ching-Yun Chang, Timothy R. Holzer, Lin Zhang, Sarah M. Engle, Stephanie Beasley, Thompson N. Doman, Lynn Naughton, Richard E. Higgs, Nicole Kallewaard, Robert J. Benschop

**Affiliations:** Eli Lilly and Company, Lilly Corporate Center, 893 S Delaware St., Indianapolis, IN 46285 USA

**Keywords:** Biomarkers, COVID-19, Gene expression, Bamlanivimab, Luminex, RNA-seq, Analytes

## Abstract

**Background:**

A thorough understanding of a patient’s inflammatory response to Severe Acute Respiratory Syndrome Coronavirus 2 (SARS-CoV-2) infection is crucial to discerning the associated, underlying immunological processes and to the selection and implementation of treatment strategies. Defining peripheral blood biomarkers relevant to SARS-CoV-2 infection is fundamental to detecting and monitoring this systemic disease. This safety-focused study aims to monitor and characterize the immune response to SARS-CoV-2 infection via analysis of peripheral blood and nasopharyngeal swab samples obtained from patients hospitalized with Coronavirus disease 2019 (COVID-19), in the presence or absence of bamlanivimab treatment.

**Methods:**

23 patients hospitalized with COVID-19 were randomized to receive a single dose of the neutralizing monoclonal antibody, bamlanivimab (700 mg, 2800 mg or 7000 mg) or placebo, at study initiation (Clinical Trial; NCT04411628). Serum samples and nasopharyngeal swabs were collected at multiple time points over 1 month. A Proximity Extension Array was used to detect inflammatory profiles from protein biomarkers in the serum of hospitalized COVID-19 patients relative to age/sex-matched healthy controls. RNA sequencing was performed on nasopharyngeal swabs. A Luminex serology assay and Elecsys® Anti-SARS-CoV-2 immunoassay were used to detect endogenous antibody formation and to monitor seroconversion in each cohort over time. A mixed model for repeated measures approach was used to analyze changes in serology and serum proteins over time.

**Results:**

Levels of IL-6, CXCL10, CXCL11, IFNγ and MCP-3 were > fourfold higher in the serum of patients with COVID-19 versus healthy controls and linked with observations of inflammatory and viral-induced interferon response genes detected in nasopharyngeal swab samples from the same patients. While IgA and IgM titers peaked around 7 days post-dose, IgG titers remained high, even after 28 days. Changes in biomarkers over time were not significantly different between the bamlanivimab and placebo groups.

**Conclusions:**

Similarities observed between nasopharyngeal gene expression patterns and peripheral blood biomarker profiles reveal a connection between the circulation and processes in the nasopharyngeal cavity, reinforcing the potential utility of systemic blood biomarker profiling for therapeutic monitoring of patient response. Serological antibody responses in patients correlated closely with reductions in the COVID-19 inflammatory protein biomarker signature. Bamlanivimab did not affect the biomarker dynamics in this hospitalized patient population.

**Supplementary Information:**

The online version contains supplementary material available at 10.1186/s12967-022-03345-3.

## Background

The Coronavirus Disease 2019 (COVID-19) pandemic has claimed the lives of over 5.7 million people worldwide, with close to 402 million cases of the disease reported to date [1]. The medical and scientific communities continue to investigate the systemic, inflammatory nature of COVID-19 infection as a prognostic measure of disease severity, in order to fully comprehend the virulence of the causative agent of COVID-19, severe acute respiratory syndrome coronavirus 2 (SARS-CoV-2). These investigations have been expedited by the emergence and prevalence of viral variants [2–4] and have prompted the rapid development and analysis of targeted therapeutic treatment options for patients suffering from advanced stages of COVID-19 infection and the resultant dysregulated immune responses [5–10].

Type I Interferons (IFN-1) play a crucial role in the host immune response to viral infections [11]. IFN-1 production is triggered by host recognition of viral nucleic acids [12], which in turn induces an antiviral signaling cascade and the expression of hundreds of genes involved in functions ranging from inhibition of viral replication to activation of immune cells [13, 14]. However, SARS-CoV viruses have the ability to delay and inhibit IFN-1 responses and IFN-activated genes, thus interfering with the normal immune response to viral infection [15–20]. This interference in IFN-mediated responses is reflected in the immunopathology of infection, ultimately leading to an exaggerated immune response in the form of large-scale pro-inflammatory cytokine production, often referred to as a cytokine storm [21, 22]. The dysregulated immune response and hyperinflammation associated with a cytokine storm contribute to disease severity in COVID-19 [23, 24]. In fact, recent studies have shown that individuals with errors in the IFN-1 pathway or neutralizing autoantibodies against IFN-1s had a greater chance of developing life-threatening COVID-19 [25, 26]. Therefore, a thorough understanding of the functional consequences of the endogenous immune response to SARS-CoV-2 infection is crucial throughout the disease process and during recovery.

Treatment with monoclonal antibodies (mAbs) represents a targeted and fast-acting therapeutic approach to diminish viral dissemination in patients with COVID-19. Bamlanivimab is a potent, neutralizing mAb that binds to the receptor binding domain (RBD) of the COVID-19 spike protein, blocking viral entry to host cells, thus preventing infection and viral replication [27]. Neutralizing mAbs, such as bamlanivimab, offer immediate passive immunity to patients, provided treatment is administered within 10 days of COVID-symptom onset [28, 29].

In the current study, we sought to detect and track the immune response to SARS-CoV-2 in patients hospitalized with COVID-19, in the presence or absence of bamlanivimab treatment, by following changes in immune-relevant parameters over the course of 1 month. We present herein, various longitudinal immunological measures within the blood of patients with COVID-19 and demonstrate the connectivity of these measurements to RNA profiles in the nasopharyngeal cavity.

## Methods

### Patients, sample information and clinical laboratory tests

23 patients hospitalized with COVID-19 were randomized at study initiation to intravenously receive one dose of 700 mg, 2800 mg or 7000 mg bamlanivimab, or placebo (J2W-MC-PYAA Clinical Trial; NCT04411628) [30]. A sentinel dosing strategy was implemented for each dose, where the first 2 patients in each cohort were randomized (1:1) to bamlanivimab and placebo. Safety and tolerability were assessed at 24 h, and subsequent participants randomized to the remaining treatment groups (5 to bamlanivimab and 1 to placebo). Infusion times varied depending on the dose received. All patients had COVID-19 symptom onset within 24 days prior to dosing and had been diagnosed with COVID-19 within 14 days prior to dosing. Baseline sequencing data was available for 14/23 of the patients. Genotypic analysis confirmed that all infecting viruses contained the D614G substitution found in the ancestral B.1 pangolin lineage. Patient serum and nasopharyngeal swabs were collected on Days 1, 3, 7, 11, 15, 22, and 29 post-infusion with follow-up sampling for nasopharyngeal swab assessments on a case-by-case basis. Clinical laboratory measurements for hematology, clinical chemistry (e.g. albumin), and viral severity panels (e.g., C-reactive protein) were assayed by local laboratories. Serum samples from 25, non-treated, age/sex-matched individuals were separately collected for baseline comparison**.**

### Olink proximity extension array (PEA)

Serum was analyzed with the Olink PEA technology platform using the Inflammation I (95302) and Cardiovascular II (95500) Proseek panels, according to manufacturer protocols. The Fluidigm BioMark HD was used to measure analyte-specific, DNA-amplified products for each sample. The unit of measure, calculated from cycle threshold (Ct) values and expressed on a log2 scale, is represented as normalized protein expression (NPX) values.

### Luminex multiplexing

Luminex serology samples were assayed as previously described [30]. Briefly, drug-tolerant SARS-CoV-2 proteins (nucleocapsid [NCP] and N-terminal domain [NTD]), to which bamlanivimab does not bind, were conjugated to MAGPlex microspheres (#MC100XX, Luminex) and prepared at 50 microspheres/µL in PBS-HS for each immunoglobulin isotype reaction. Serum samples were centrifuged at 10,000×*g* for 5 min at 4 °C and diluted 1:400 in phosphate buffered saline-high salt solution (PBS-HS; 0.01 M PBS, 1% BSA, 0.02% Tween, 300 mM NaCl) and combined into a prewet assay plate (#3632; Corning) for each isotype. Plates were protected from light while shaking at 450 rpm for 90 min at 22 °C. After washing three times with PBS-HS the secondary phycoerythrin-conjugated antibody (αIgG, #109-115-098, Jackson Labs; αIgM, #709-116-073, Jackson Labs; αIgA, #109-115-011, Jackson Labs) was added to the appropriate plate and incubated shaking at 450 rpm for 30 min at 22 °C. Plates were washed (3×) with PBS–low salt solution (PBS-LS; 0.01 M PBS, 1% BSA, 0.02% Tween) and samples resuspended in PBS-LS and read using a Luminex 200 System (#APX10031; Luminex) with xPONENT Software.

### Elecsys® anti-SARS-CoV-2 immunoassay

Elecsys® Anti-SARS-CoV-2 (Roche Diagnostics) is an immunoassay intended for the qualitative detection of antibodies to SARS-CoV-2 in human serum and plasma. The assay, performed per manufacturer’s directions, uses a recombinant protein representing the NCP antigen for the determination of antibodies against SARS-CoV-2. The test is intended for use as an aid in identifying individuals with an adaptive immune response to SARS-CoV-2, indicating recent or prior infection. This test was approved for use under Emergency Use Authorization (EUA) and analytically verified in an accredited laboratory in compliance with the Clinical Laboratory Improvement Amendments (CLIA) by the College of American Pathologists (CAP). Test samples were assessed using a Roche Cobas E Analyzer.

### Targeted RNA-Seq

Nasopharyngeal swabs were collected in viral transport media (VTM). Nucleic acid was extracted from 200 µL VTM using MagMAX Viral/Pathogen Binding Beads (ThermoFisher Scientific) on a Microlab STAR (Hamilton). Reverse transcription was performed using SuperScript VILO cDNA Synthesis kit (Thermo). A custom Ion AmpliSeq RNA panel (Thermo) was used for Next-Generation Sequencing (NGS) library preparation on the Ion Chef system (Thermo). The panel comprised 798 inflammation-related genes matching 1617 NCBI accessions (Additional file 2: spreadsheet). Templating was performed using Ion 540 chips. Chips were sequenced on the Ion Torrent S5XL (Thermo). Gene expression data was subjected to QC steps including the rejection of any samples with fewer than 500k total read counts. Lowly expressed genes were flagged with the R function filterByExpr in the edgeR library [31]. A total of 87 genes were filtered out by this method, leaving 709 genes for analysis. Gene expression count data was normalized as reads per million (RPM). And then differentially expressed genes were identified using Likelihood Ratio Test (LRT) within the DeSeq2 package [20] using a p-value threshold of 0.05. These were analyzed within the Broad Institute “Investigate Gene Sets” computational protocol (http://www.gsea-msigdb.org/gsea/login.jsp) to identify over-represented genes. The differentially expressed genes were clustered using K-means in Spotfire Analyst (TIBCO, version 10.10.3).

### Statistical analyses

#### Baseline protein profiles from serum of patients with COVID-19

For each marker, an analysis of variance (ANOVA) model of patients with COVID-19 and healthy volunteers on protein concentration NPX was applied. For across markers multiple comparison adjustment, Benjamin-Hochberg correction was used, and fold change and adjusted p-values were reported. A volcano plot with the log2 fold change of patients with COVID-19 to healthy volunteers was plotted with corresponding adjusted p-values. The significant up-/down-regulated markers are colored in red/blue, as appropriate (i.e., fold change > 1.5 and adjusted p-values < 0.05).

#### Pharmacodynamic analysis of serum protein changes over time

For each marker, a MMRM (mixed model for repeated measures) with treatment and time variables was applied to protein concentration NPX. Contrasts of pharmacodynamic change of day 3, 7, 11, 15, 22, 29 to baseline compared with bamlanivimab and placebo were applied with multiplicity adjustment using Benjamini and Hochberg method at the significant threshold of 0.05 [nlme package in R]. A mean profile plot was generated with the mean and standard error of each treatment arm over time, as well as the mean of healthy volunteer protein concentration level colored in blue for benchmark.

#### Pharmacodynamic analysis of serology over time

For each marker, MMRM with treatment and time variables was applied to serology concentration. Contrasts of pharmacodynamic change of day 3, 7, 11, 15, 22, 29 to baseline compared with IgA and IgM to IgG were applied with multiplicity adjustment using Benjamini and Hochberg method at the significant threshold of 0.05 [nlme package in R]. A mean profile plot was generated with the mean and standard error of each treatment arm over time. Individual patient plot was provided for each treatment and highlighted with Cobas assay status (negative, positive, and non-detected).

#### Titer estimation

Titer was estimated using the two dilution points that straddle the titer cut point factor. If the maximum signal of a titration curve is less than the cut point, then the titer is imputed as 800 (the smallest dilution).

### Viral load assessment

Viral load samples collected by either nasopharyngeal swab or mid-turbinate swab were tested for the presence of SARS-CoV-2 as previously described [30]. Briefly, the SARS-CoV-2 viral load was derived from the Ct values determined using a reverse transcription polymerase chain reaction assay of viral genes N1 and N220; Ct values ranged between 0 and 45, where higher Ct values indicated a lower viral load21. Where Ct was determined (a positive test result), viral load was derived as follows: Viral load = (Cutoff-CtNi)/(log210). Where Cutoff is 45 for statistical analysis (other values, such as 40 for PK/PD analysis were explored); i = 1 or 2. Ct for N1 was used as the primary measure; Ct for N2 was only used when Ct for N1 was not available. Viral load results are presented in log base 10 scale.

## Results

### Olink proteomic characterization of serum of patients hospitalized with COVID-19

**Fig. 1:**
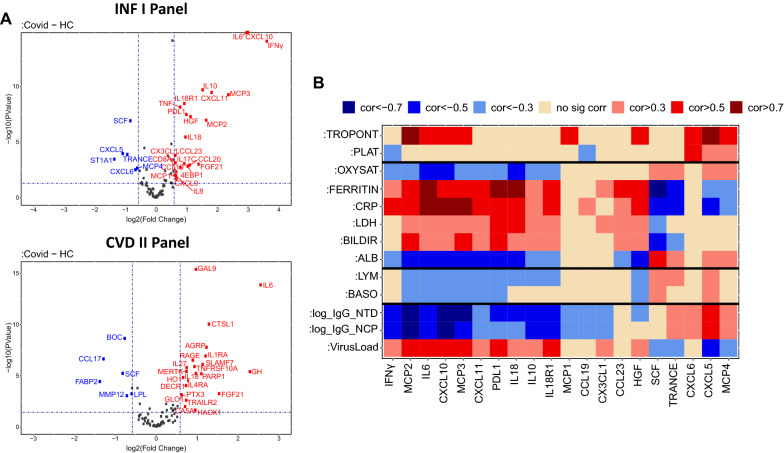
Characterization of proteome serum measurements, taken from patients hospitalized with COVID-19. **a** Volcano plots demonstrating Olink Proteomic Analysis (Inflammation I (top) and Cardiovascular II (bottom)) from baseline measurements taken from patients with COVID-19 vs age/sex-matched healthy controls (HC). Proteins lower in expression in patients with COVID-19 are indicated in blue, more highly expressed proteins are highlighted in red. **b** Heatmap showing repeated measures correlations from longitudinal patient samples of key protein biomarkers to viral load, clinical readouts (e.g., OXYSAT, Ferritin (refer to abbreviations list for others)), and cell counts. The color value of the cells is proportional to the strength of the associations as indicated in the color scale (top of the panel) and ranges from negative (blue) to positive (orange/red)

Olink PEA analysis of serum samples taken from patients hospitalized with COVID-19 revealed obvious circulating inflammatory profiles compared to samples taken from age/sex-matched healthy controls. Protein biomarker panels which focused on inflammatory and cardiovascular-linked processes (Fig. 1A), revealed many of the inflammatory markers identified are linked to interferon (IFN) signaling pathways, which play a vital role in the immune response to viral infection. The most significantly elevated levels in the serum of patients with COVID-19 relative to matched healthy controls were interleukin (IL) 6; IL-6 (fold change (FC) = 7.8), C–X–C motif chemokine ligand 10; CXCL10 (FC = 8.0), CXCL11 (FC = 3.5), IFNγ (FC = 12.2), and Monocyte Chemotactic Protein-3; MCP-3 (FC = 5.1) (Fig. 1A).

Interestingly, there was a significant inverse correlation (p < 0.05) between many of these dysregulated protein biomarkers (e.g., IL-6) with clinical measures, such as lymphocyte count (LYM, corr = − 0.34, p < 0.05), albumin (ALB, corr = − 0.62 p < 0.05), and oxygen saturation (OXYSAT, corr = − 0.51 p < 0.05), while a positive link (corr > 0.7 p < 0.05) existed to known inflammatory measures, ferritin and C-Reactive Protein (CRP) (Fig. 1B, Additional file 1: Fig. S1). In similar fashion, protein biomarkers reduced in the circulation of COVID-19 patients relative to age/sex-matched healthy controls (i.e., Stem Cell Factor (SCF) and Tumor Necrosis Factor-related Activation induced Cytokines (TRANCE), were inversely correlated with traditional inflammatory measures such as ferritin and CRP (Fig. 1A, B). These key markers were not only differentially regulated in the serum of patients with COVID-19 vs healthy controls, but importantly, correlated with viral load levels from nasopharyngeal swab PCR testing (Fig. 1B).

#### Longitudinal assessment of inflammatory protein mediators

**Fig. 2:**
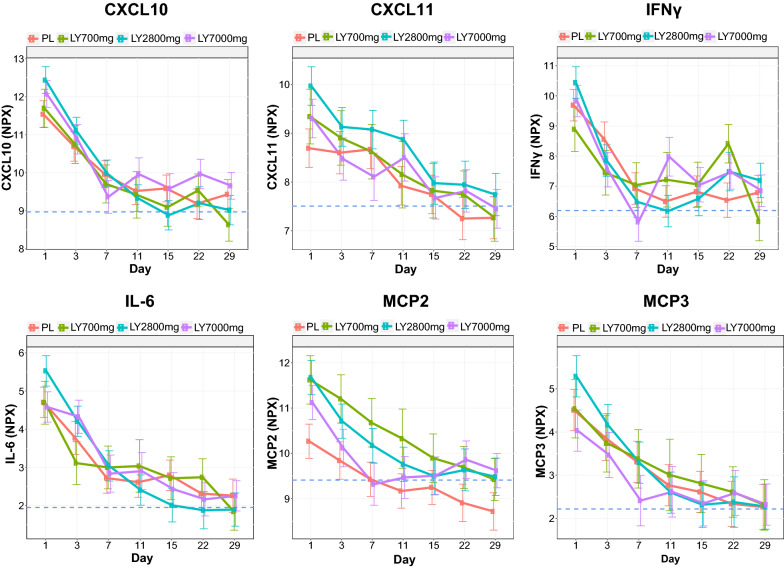
CXCL10, CXCL11, IFNγ, IL-6, MCP2, and MCP3 time-dependent response curves. Dashed line (---) represents circulating levels of inflammatory proteins in healthy controls. Treatment cohorts are represented as PL (placebo), LY700mg (bamlanivimab 700 mg dose), LY2800mg (bamlanivimab 2800 mg dose) and LY7000mg (bamlanivimab 7000 mg dose) with corresponding colors

As shown, numerous inflammatory mediators were upregulated at baseline in patients with COVID-19, relative to healthy controls. Over time, most mediators reverted back to healthy control levels as patients recovered from COVID-19. A decrease in IFNγ and CXCL10 was observed in patients as early as Day 3 whereas others (e.g., CXCL11) took longer to return to normal (Fig. 2). Levels of several inflammatory analytes that were highly correlated to viral load, such as IL-6, CXCL11, MCP2, and MCP3 (Fig. 1), began returning to the range observed in the circulation of age/sex-matched healthy controls by Days 7–11 (Fig. 2). No statistically significant difference was observed between the four treatment cohorts of this small study. All treatment cohorts (including placebo) demonstrated resolution of dysregulated inflammatory markers by returning to the range observed within healthy control samples.

#### Antibody production in serum of COVID-19 patients

**Fig. 3:**
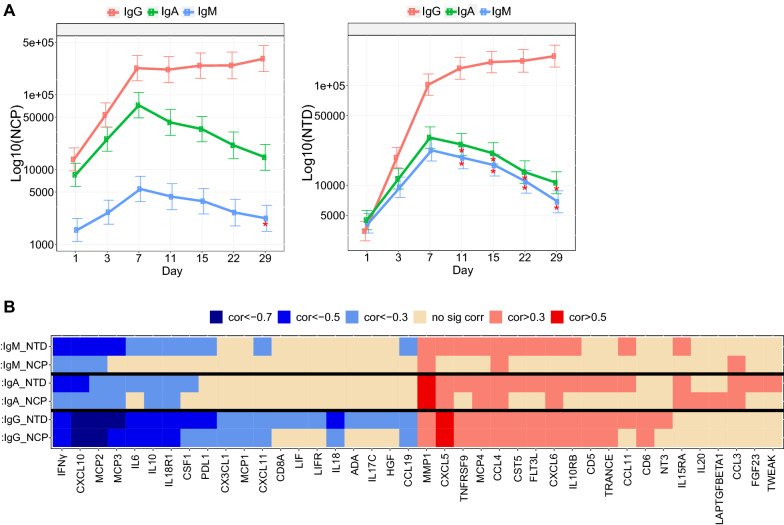
Luminex Serology Assay. **a** All patients (placebo and treatments) graphed with mean (+ SE) for IgA, IgG and IgM endogenous antibody formation against the NCP and NTD. Time dependent changes in serology compared to IgG at the same timepoint are highlighted with a star. **b** Heatmap showing repeated measures correlations from longitudinal patient samples of inflammatory protein biomarkers to N-terminal domain (NTD) and nucleocapsid (NCP). The color value of the cells is proportional to the strength of the associations as indicated in the color scale (top of the panel) and ranges from negative (blue) to positive (orange/red), and p-value < 0.1

Endogenous antibody formation of major immunoglobulin (Ig) serotypes (IgA, IgG, IgM) was determined using SARS-CoV-2 antigens that are unaffected by bamlanivimab treatment, including NCP and NTD using a Luminex Serology Assay (Fig. 3A). As expected, titers increase over time peak 7 days from study entry. A similar time course of induction was observed for the IgA, IgG and IgM isotypes.

Correlations were identified between antibody titers from all antibody classes tested and the change in levels of inflammatory mediators in patient serum samples over the course of 29 days of treatment (Fig. 3B). IL-6, Monocyte chemoattractant protein-2 (MCP-2), MCP-3 and CXCL10 all showed strong inverse correlations, many between − 0.5 and − 0.7. Matrix Metallopeptidase 1 (MMP-1) and CXCL5 were moderately or strongly correlated (r = 0.5–0.7). Since all antibody classes performed similarly in these analyses, further analysis focused on IgG titers.

#### Longitudinal assessment of IgG binding to the NCP and NTD domains

Longitudinal analysis of IgG titers demonstrated an increase in endogenous IgG antibodies against SARS-CoV-2 NCP protein reaching plateau between day 7 through 29 for each treatment group (Fig. 3A). Analysis of seroconversion (the development of specific antibodies in the blood, directed against an infectious agent) towards NCP using the Elecsys® Anti-SARS-CoV-2 Assay and Cobas Analyzer from Roche provided additional confirmation of seroconversion, however, with less sensitivity than the Luminex method (Additional file 1: Fig. S2).

#### Linking serum cytokine levels and nasopharyngeal swab gene expression

**Fig. 4:**
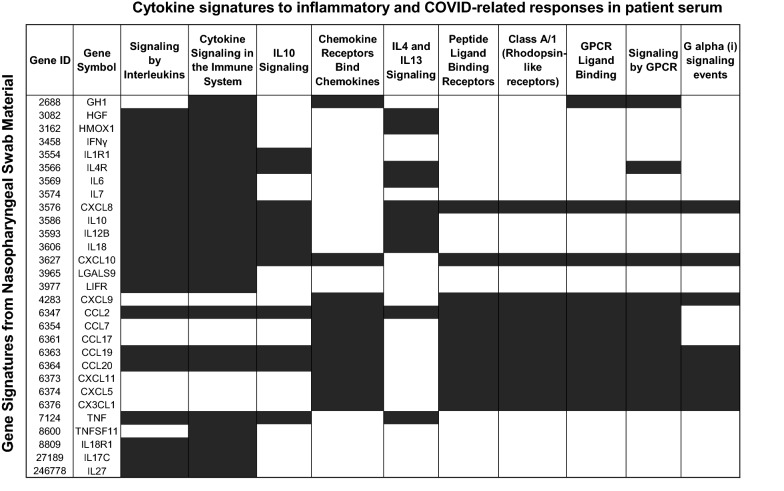
Connection between nasopharyngeal swab gene expression to observed cytokine signatures in patient serum across time points. Shaded regions highlight COVID-19-related immune response gene expression correlation between nasopharyngeal and serum samples from patients with COVID-19

Characterization of gene expression signals identified in the nasopharyngeal cavity of patients with COVID-19 revealed immune response gene expression patterns related to inflammatory proteins observed in patient serum. Figure 4 highlights the association of the genes expressed in the nasopharyngeal cavity to interferon-linked processes (signaling events and receptor activation) identified prominently in patient serum. Additionally, key inflammatory markers of interest, C–C motif chemokine ligands 2, 19, and 20 (*CCL2*, *CCL19,* and *CCL20*), C–X–C motif chemokine ligands 8 and 10 (*CXCL8* and *CXCL10*), and *IL-10*, were found in both nasopharyngeal swab material and the circulating blood of patients with COVID-19 across time points.

#### Longitudinal assessment of gene expression from nasopharyngeal swabs

**Fig. 5:**
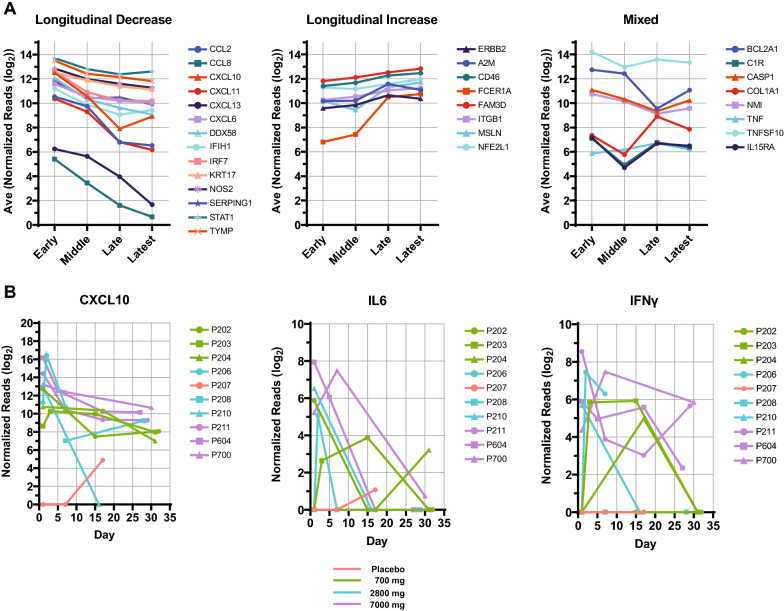
Time-dependent gene expression responses from nasopharyngeal swabs. **a** Average normalized read counts for selected gene expression markers were plotted by time and grouped by trend observed over the course of the trial. Early, day 1; Middle, days 3–11; Late, days 15–17; Latest, days ≥ 27 post-infusion with bamlanivimab. **b** *CXCL10*, *IL6*, and *IFN*γ time-dependent changes in normalized reads by patient (series labels). Series colors indicate treatment groups. Abbreviations are listed at the end of the document

Targeted RNA-Seq was used to monitor changes in inflammatory and virus-related immune response pathways shown to be dysregulated in the nasopharyngeal samples from patients with COVID-19 (Fig. 4). To enable comparisons among genes, samples were grouped based on time post-infusion: early (day 1), mid (days 3–11), late (days 15–17), latest (≥ 27 days). Analysis using the LRT revealed 30 IFN-responsive genes that were differentially expressed among timepoints. Clustering analysis enabled grouping of statistically significant results into three categories: (i) genes that showed a longitudinal decrease, (ii) a longitudinal increase, or (iii) a mixed result (Fig. 5A). We further focused on 3 highly dysregulated protein analytes observed in the circulation of patients (Fig. 1A) in order to better understand the responses occurring in the nasopharyngeal cavity of these same patients. A closer look at one of these differentially expressed genes, *CXCL10*, shows high baseline expression that trends downward in patients across all timepoints, concordant with CXCL10 protein-level assessments (Fig. 5B). Perhaps due to the rapid decrease of *IL6* gene expression in nasopharyngeal swabs, this gene was not identified as differentially expressed in longitudinal fashion between timepoint groups (Fig. 5A); however, the trend was concordant with changes observed in IL-6 protein analyses. Comparison of mRNA expression of *IFN*γ with IFNγ protein assessment shows both trending toward lower levels of expression over the course of the study (Fig. 5B). Together, the similarities between the longitudinal gene expression in the nasopharyngeal cavity and the longitudinal circulating cytokine protein analyses are consistent with the idea that the circulating markers reflect the biology at the site of infection.

## Discussion

The uncontrolled release of pro-inflammatory cytokines and the cytokine storm which ensues has been recognized as the primary cause of death in patients infected with SARS-CoV-2 [32]. Proteomic analysis highlighted a cytokine storm in the serum of patients with COVID-19, with elevated expression levels of IL-6, CXCL10, CXCL11, IFNγ, MCP-2, and MCP-3 detected in infected patients relative to healthy controls. Many inflammatory markers identified using this analysis were associated with IFN signaling, and an interesting correlation was observed between ferritin and CRP levels with IL-6 and CXCL10. Elevated levels of CRP, ferritin, and IL-6 concentrations have been shown to be higher in cytokine storm disorders [33], patients with severe COVID-19, and in non-survivors [34, 35] of COVID-19 compared to discharged patients. In addition, previous studies support our observations that concentrations of these biomarkers decreased as patients recover [36]. Lastly, these same biomarkers may be critically important indicators of patient outcome as elevated clinical inflammatory markers were inversely correlated with OXYSAT levels and tracked with lymphopenia responses observed in non-survivors of COVID-19 [37]. This first-in-man study of bamlanivimab was not powered to detect any effects of treatment on longitudinal changes in immune biomarkers and no significant effects were observed.

As we looked to better understand the lymphocyte responses over time in these patients, we focused on serological assays, which demonstrate a high degree of sensitivity and specificity, and are highly favored for the detection and monitoring of anti-SARS-CoV-2 antibodies in patients who present with COVID-19. The accuracy of these assays is significant to clinical management and public health decision-making. In the current study, we demonstrate use of the Luminex serology assay to detect quantitative seroconversion in clinical specimens from patients with COVID-19 with greater sensitivity than the Elecsys® Anti-SARS-CoV-2 assay. While the Elecsys® Anti-SARS-CoV-2 assay is rapid [38] and has received FDA EUA approval [39], the Luminex method holds many advantages due to the ability to ascertain broad serological conversion toward multiple viral proteins, including drug-tolerant epitopes, at the same time. A robust antibody response to the drug-tolerant, NCP and NTD of the SARS-CoV-2 spike protein was observed using this assay, with high titers of IgA, IgG, and IgM sharing a similar time course of induction. While IgA and IgM titers peaked around 7 days post-dose, IgG titers remained high, even after 28 days. These findings are in accordance with previous observations made across a number of studies [40–42], and highlight the utility of the Luminex assay for detection and monitoring of anti-SARS-CoV-2 antibodies.

The inverse correlation observed between the levels of antibodies produced against the NCP and NTD of the SARS-CoV-2 spike protein and the levels of inflammatory mediators present in serum samples is indicative of the power of humoral immunity to resolve infection. The ability of the Luminex serology method to detect both bamlanivimab as well as the endogenous immune response towards spike protein has already been documented [43]. There were no observed changes in the endogenous antibody responses in bamlanivimab-treated patients vs placebo in this small study, although larger studies are ongoing. The lack of distinguishable biomarker differentiation between placebo and bamlanivimab arms was not unexpected based on subsequent studies demonstrating that passive immunization was not an effective treatment for patients hospitalized with COVID-19 [44–46].

Importantly, our study revealed a remarkably similar pattern in gene expression profiles observed in nasopharyngeal swab material and the inflammatory, COVID-19-related responses observed in the serum of infected patients. While responses were not different between bamlanivimab- treated individuals and those who received placebo, *CCL2*, *CCL19, CCL20, CXCL8* and *CXCL10* were upregulated in samples from the nasopharyngeal cavity and the circulating blood of patients with COVID-19. Many of these chemokines are strongly linked to macrophage and neutrophil function. *CCL2* is vital for monocyte recruitment [29], is expressed at higher levels in lung macrophages of patients with severe COVID-19 [30] and upregulated in response to increases in SARS-CoV-2 viral load levels in infected patients [31]. *IL-8/CXCL8* plays a key role in the recruitment and activation of neutrophils during inflammation [33]. Given that neutrophilia is frequently observed in patients with COVID-19 [47–49], it is possible that *CXCL8* contributes to the pathophysiology of the disease. The elevated expression levels of *CXCL8,* which we observed in the serum of patients with COVID-19, reflected the findings of Del Valle et al. in their study examining predictive biomarkers of SARS-CoV-2-related inflammation [50]. Our study aligns with the authors observations that high levels of *CXCL8* were associated with worse patient outcomes and may be an important predictive biomarker of patient survival. Similarly, Li et al. revealed that high levels of *CXCL8* in the serum of patients with COVID-19 were consistent with severity of disease [51].

In line with recent bioinformatic analysis of COVID-19 sequencing data we determined elevated expression of IFNγ -linked biomarkers, such as CXCL10, in both serum and nasopharyngeal swab material collected from patients with COVID-19. While this study was small and failed to identify pharmacodynamic biomarkers for bamlanivimab, the rapid decrease of key inflammatory mediators (e.g., IFNγ, IL-6, IL-10, MCP-2, MCP-3, and CXCL10), beginning as early as Day 3, emphasizes the need for normalization of key immune analytes for favorable outcomes in the course of COVID-19 infection. These observations add to our understanding of core biomarkers of COVID-19 severity, including recent observations in children suffering from multisystem inflammatory syndrome, and further our knowledge around key immune pathways that therapeutic options must target early in infection to best promote favorable patient outcomes [52]. This could be most valuable when evaluating the pathology of SARS-CoV-2 variants or when performing combination studies of multiple neutralizing mAbs. Future studies could be focused on monoclonal-resistant SARS-CoV-2 variants, with the current knowledge that serum biomarkers adequately reflect the nasal compartment.

## Conclusions

Whilst this small, safety-focused study did not provide additional mechanistic data concerning bamlanivimab’s therapeutic effects, it did reveal an important correlation between COVID-19-specific biomarker profiles from the peripheral blood and gene expression patterns from nasopharyngeal swab material taken from patients hospitalized with COVID-19. This finding gives strength to the potential utility of systemic blood biomarker profiling for therapeutic monitoring of a patient’s response to treatment. Additionally, while the Elecsys® Anti-SARS-CoV-2 can adequately and readily detect antibodies to N protein, other methods, such as the Luminex serology assay described herein are more sensitive and may support SARS-CoV-2 research in future studies.

## Supplementary Information

**Additional file 1.** Supplementary figures and methods.

**Additional file 2.** Listing of inflammation-related and COVID-19 implicated genes in custom Ion AmpliSeq RNA panel.

## Data Availability

The datasets used and/or analyzed during the current study are available from the corresponding author on reasonable request.

## Abbreviations

4EBP1: Eukaryotic translation initiation factor 4E-binding protein 1
A2M: Alpha-2-macroglobulin
ADA: Adenosine deaminase
AGRP: Agouti-related peptide
ALB: Albumin
BASO: Basophil
BCL2A1: BCL2 related protein A1
BILDIR: Direct bilirubin
BOC: Brother of CDO
C1R: Complement C1R subcomponent
CASA: Cancer associated serum antigen
CASP1: Caspase 1
CASP8: Caspase 8
CCL2: C–C motif chemokine ligand 2
CCL3: C–C motif chemokine ligand 3
CCL4: C–C motif chemokine ligand 4
CCL8: C–C motif chemokine ligand 8
CCL11: C–C motif chemokine ligand 11
CCL17: C–C motif chemokine ligand 17
CCL19: Chemokine (C–C motif) ligand 19
CCL20: Chemokine (C–C motif) ligand 20
CCL23: Chemokine (C–C motif) ligand 23
CD5: Cluster designation 5
CD6: Cluster designation 6
CD46: Cluster designation 46
CD244: Cluster designation 244
CD8A: Cluster designation 8a
CDCP1: CUB-domain-containing protein 1
COL1A1: Collagen type I alpha 1 chain
CRP: C-reactive protein
CSF1: Colony stimulating factor 1
CST5: Cystatin D
CTSL1: Cathepsin L1
CX3CL1: Serum fractalkine
CXCL1: C–X–C motif chemokine ligand 1
CXCL6: C–X–C motif chemokine ligand 6
CXCL10: C–X–C motif chemokine ligand 10
CXCL11: C–X–C motif chemokine ligand 11
CXCL13: C–X–C motif chemokine ligand 13
CXCL5: C–X–C motif chemokine ligand 5
CXCL6: C–X–C motif chemokine ligand 6
DECR1: 2,4-Dienoyl CoA reductase 1
DDX58: DExD/H-Box Helicase 58
ENRAGE: Calcium-binding protein S100A12
ERBB2: Erb-B2 receptor tyrosine kinase 2
FABP2: Fatty acid binding protein 2
FAM3D: FAM3 metabolism regulating signaling molecule D
FCER1A: Fc Fragment of IgE Receptor Ia
FGF23: Fibroblast growth factor-23
FGF21: Fibroblast growth factor 21
FLT3L: Flt-3 Ligand
GAL9: Extra-cellular galectin-9
GDNF: Glial cell line-derived neurotrophic factor
GH: Growth hormone
GLO1: Glyoxalase I
HAOX1: Hydroxyacid oxidase 1
HGF: Hepatocyte growth factor
HO1: Heme oxygenase-1
IFIH1: Interferon induced with Helicase C domain 1
IFNγ: Interferon gamma
IL15RA: Interleukin 15 receptor alpha
IL1ALPHA: Interleukin 1 alpha
IL1RA: Interleukin-1 receptor antagonist
IL2: Interleukin 2
IL4RA: Interleukin 4 receptor subunit alpha
IL6: Interleukin 6
IL10: Interleukin 10
IL10RA: Interleukin 10 receptor subunit alpha
IL10RB: Interleukin 10 receptor subunit beta
IL17C: Interleukin 17C
IL18: Interleukin 18
IL18R1: Interleukin 18 receptor 1
IL20: Interleukin 20
IL27: Interleukin 27
IRF7: Interferon regulatory factor 7
ITGB1: Integrin Subunit Beta 1
KRT17: Keratin 17
LAPTGFBETA1: Transforming growth factor beta-1
LDH: Lactate dehydrogenase
LIF: Leukemia inhibitory factor
LIFR: Leukemia inhibitory factor receptor
LYM: Lymphocyte
MCP1: Monocyte chemoattractant protein-1
MCP2: Monocyte chemoattractant protein-2
MCP3: Monocyte chemoattractant protein-3
MCP4: Monocyte chemoattractant protein-4
MERTK: Membrane-bound receptor tyrosine kinase
MMP-1: Matrix metallopeptidase 1
MMP-12: Matrix metallopeptidase 12
MSLN: Mesothelin
NCP: Nucleocapsid protein
NFE2L1: Nuclear factor, erythroid 2 like 1
NMI: N-Myc and STAT interactor
NT3: Neurotrophin 3
NOS2: Nitric oxide synthase 2
OSM: Oncostatin M
OXYSAT: Oxygen saturation
P202: Interferon activated gene 202
P203: Interferon activated gene 203
P204: Interferon activated gene 204
P206: Interferon activated gene 206
P207: Interferon activated gene 207
P208: Interferon activated gene 208
P210: Interferon activated gene 210
P211: Interferon activated gene 211
P604: Interferon activated gene 604
P700: Interferon activated gene 700
PARP1: Poly(ADP-Ribose) polymerase 1
PDL1: Programmed death-ligand 1
PLAT: Platelets
PTX3: Pentraxin 3
RAGE: Receptor for advanced glycation end products
SCF: Serum stem cell factor
SERPING1: Serpin Family G Member 1
SLAMF7: Signaling lymphocytic activation molecule family 7
ST1A1: Sulfotransferase 1A1
STAT1: Signal transducer and activator of transcription 1
TNF: Tumor necrosis factor
TNFRSF9: Tumor necrosis factor receptor superfamily member 9
TNFRSF10A: Tumor necrosis factor receptor superfamily member 10A
TNFSF10: Tumor necrosis factor superfamily member
TNFSF14: Tumor necrosis factor superfamily member 14
TRAILR2: Tumor necrosis factor-related apoptosis-inducing ligand receptor 2
TRANCE: Tumor necrosis factor-related activation induced cytokines
TROPONT: Troponin T
TWEAK: Tumor necrosis factor-like weak inducer of apoptosis
TYMP: Thymidine phosphorylase
