# Supplementary material for: Relationship between gene expression patterns from nasopharyngeal swabs and serum biomarkers in patients hospitalized with COVID-19, following treatment with the neutralizing monoclonal antibody bamlanivimab
Source: J Transl Med. 2022 Mar 18;20:134. doi: 10.1186/s12967-022-03345-3 (PMC8931785; doi:10.1186/s12967-022-03345-3)
Supplement: Supplementary file 1 — Additional file 1. Supplementary figures and methods. [file 12967_2022_3345_MOESM1_ESM.docx]

**Supplementary Material**

**Supplementary figures and methods** pgs 2-3

**Spreadsheet containing inflammation-related genes and COVID-19 implicated genes used in custom Ion AmpliSeq RNA panel** provided to journal


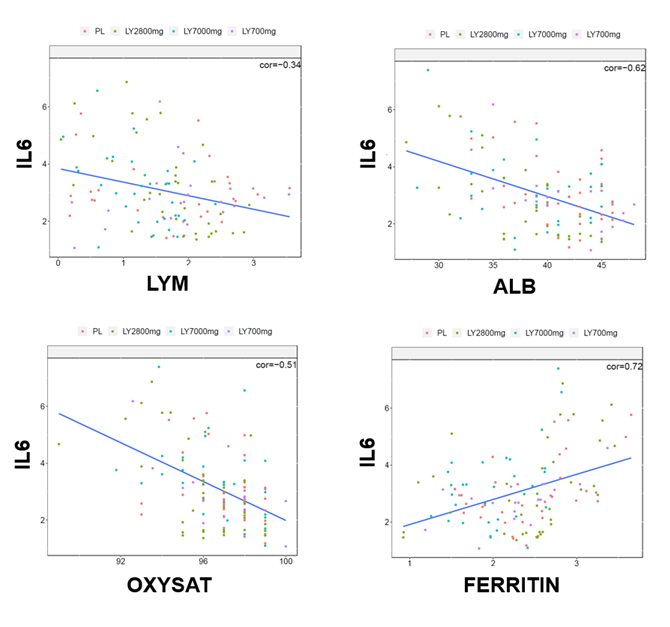


**Figure S1:** Dot plots demonstrating correlations of key inflammatory markers to clinical measures.

**
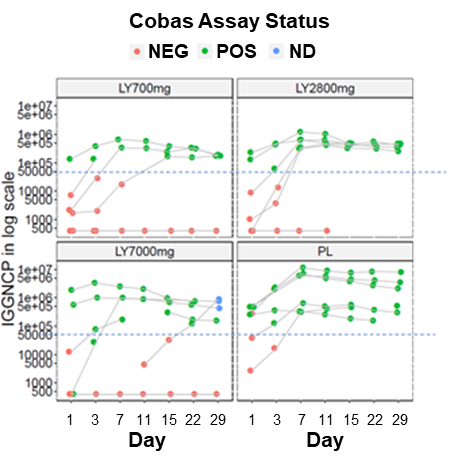
**

**Figure S2:** Time dependent responses across biomarkers. Comparison of anti-NCP IgG antibody detection across patient cohorts using the Luminex serology assay relative to results obtained using the Roche Cobas Analyzer for Elecsys® immunoassay sample assessment. Red circles indicate the same sample was negative on the Cobas Analyzer. Green circles indicate a sample’s positivity on the Cobas Analyzer. Blue circles indicate samples with no analogous Cobas data point.

Figure S2 represents anti-NCP IgG antibody detection across patient cohorts using the Luminex serology assay. Results are color coded to highlight samples which were negative (red) or positive (green) when run on the Cobas Analyzer (blue indicates no sample data was available using Cobas). The results presented demonstrate that most Cobas-positive samples lie above an arbitrary cut-off point near an anti-NCP IgG titer of 50,000, indicating higher sensitivity is achieved using the Luminex method. Most patient samples were seropositive by both methods by day 15, in line with previously reported 97.1% positivity ≥ 14 days after PCR confirmation using the Elecsys® Anti-SARS-CoV-2 Assay (52). No significant delay in endogenous IgG antibody production was observed for any of the bamlanivimab concentrations examined, compared to placebo (30).
